# Neonicotinoids and ectoparasitic mites synergistically impact honeybees

**DOI:** 10.1038/s41598-019-44207-1

**Published:** 2019-06-04

**Authors:** Lars Straub, Geoffrey R. Williams, Beatriz Vidondo, Kitiphong Khongphinitbunjong, Gina Retschnig, Annette Schneeberger, Panuwan Chantawannakul, Vincent Dietemann, Peter Neumann

**Affiliations:** 1 Institute of Bee Health, Vetsuisse Faculty, University of Bern, Bern, Switzerland; 2 Swiss Bee Research Centre, Agroscope, Bern, Switzerland; 3 Department of Entomology and Plant Pathology, Auburn University, Auburn, AL USA; 4 Veterinary Public Health Institute, Vetsuisse Faculty, University of Bern, Bern, Switzerland; 5 Bee Protection Laboratory, Department of Biology, Faculty of Science, Chiang Mai University, Chiang Mai, Thailand; 6 School of Science, Mae Fah Luang University, Chiang Rai, Thailand; 7 Environmental Science Research Center, Faculty of Science, Chiang Mai University, Chiang Mai, 50200 Thailand; 8 Department of Ecology and Evolution, University of Lausanne, 1015 Lausanne, Switzerland

**Keywords:** Conservation biology, Invasive species, Invasive species

## Abstract

The Western honeybee, *Apis mellifera*, is the most important managed pollinator globally and has recently experienced unsustainably high colony losses. Synergistic interactions among stressors are believed to be primarily responsible. However, despite clear evidence of strong effect on honeybee longevity of widely-employed neonicotinoid insecticides and of the ubiquitous ectoparasitic mite *Varroa destructor*, no data exist to show synergistic effects between these two stressors. Even though neonicotinoids had no significant impact by themselves, we here show for the first time a synergistic time-lag interaction between mites and neonicotinoids that resulted in significantly reduced survival of long-lived winter honeybees. Even though these mites are potent vectors of viruses, the virus-insecticide interaction had no significant impact. The data suggest a previously overlooked mechanism possibly explaining recent unsustainably high losses of managed *A*. *mellifera* honeybee colonies in many regions of the world. Future mitigation efforts should concentrate on developing sustainable agro-ecosystem management schemes that incorporate reduced use of neonicotinoids and sustainable solutions for *V*. *destructor* mites.

## Introduction

As pollinators, bees provide a key ecosystem service that is essential for human food security and maintenance of natural biodiversity^1,2^. The Western honeybee, *Apis mellifera*, is the single most important managed pollinator globally^3^. Therefore, unsustainably high colony mortality experienced by *A*. *mellifera* beekeepers in many countries is alarming^4^.

Interactions among stressors are believed to be primarily responsible for increased *A*. *mellifera* colony mortality recently observed^5,6^. It must be pointed out that such proposed interactions have to yield strong synergistic, or at least additive effects, to have a significant impact on a global scale. To date, comparatively little attention has focussed on the combined effects of two confirmed *A*. *mellifera* stressors – the mite *Varroa destructor* and neonicotinoid insecticides. The former is a nearly ubiquitous ectoparasite of Asian origin that is the main biological cause of honeybee colony mortality worldwide^4,7^, primarily because of viral infections vectored by this parasite^8^. The neonicotinoids are among the most commonly employed insecticides globally and are known to elicit wide-ranging lethal and sublethal effects on honeybees^9,10^. The effects of *V*. *destructor*, viruses and neonicotinoids have been intensively studied independently over the past decades^7,10,11^. Even though the co-occurrence between *V*. *destructor* and neonicotinoids is virtually inevitable in honeybee colonies^12^, possible effects of their interactions have rarely been considered. The few previous studies on the topic have not shown synergistic interactions between these mites and insecticides^13–15^, even though those are often assumed to occur. Moreover, the timing of interactions needs to be taken into account. Indeed, in autumn, colonies produce the usually long-living winter honeybees, which are essential for survival in temperate regions^16,17^. Therefore, reduced longevity of the winter bees would increase risks for colony death. Besides longevity, previous studies have shown that pathogens and neonicotinoid insecticides can affect adult emergence mass^15,18^. Since body mass is a proxy for individual performance (e.g. thermoregulation^19^), this sublethal effect may also yield consequences at the honeybee colony level. To this date, the impact of combined neonicotinoid exposure and *V*. *destructor* mite parasitism on winter honeybee weight and life expectancy has not been addressed. Similarly, the reported interactions between neonicotinoids and viruses^20^ have not been verified in field colonies yet, even though the proportion of honeybee workers with clinical symptoms of deformed wing virus (DWV) is a known predictive marker for colony collapse^21^. Given synergistic or additive interactions between *V*. *destructor* and neonicotinoids reducing winter honeybee longevity and increasing clinical DWV symptoms, a previously overlooked key mechanism contributing to overwintering colony mortality would emerge.

In a fully crossed experiment, we investigated sublethal and lethal effects of neonicotinoids and *V*. *destructor* on individual adult honeybee workers. Using a pre-established method^22^, we exposed honeybee colonies to neonicotinoid contaminated pollen (at field-realistic doses of 4 ppb thiamethoxam and 2 ppb clothianidin) or to control uncontaminated pollen for 42 days in spring, a time when colonies are typically exposed to plant protection products^23^. Within each group of colonies, workers parasitized by *V*. *destructor* during their development or not were collected to obtain a fully crossed design. To investigate possible seasonal effects, we measured the clinical symptoms of DWV and worker mass on emergence and longevity in laboratory cages. This was conducted in summer immediately following neonicotinoid exposure and 16 weeks later in autumn when colonies rear winter bees.

## Results

### Pollen patty consumption and colony strength

Average daily consumption was measured by weighing the leftover pollen patties (median colony consumption per day was ~70–73 grams); no significant differences were observed between neonicotinoid treated and non-exposed control colonies (Extended Data Table 1). Colony strength assessments revealed no significant differences between control and neonicotinoid insecticide treatment (Extended Data Table 1).

### *V*. *destructor* infestation levels and DWV clinical symptoms

**Figure 1:**
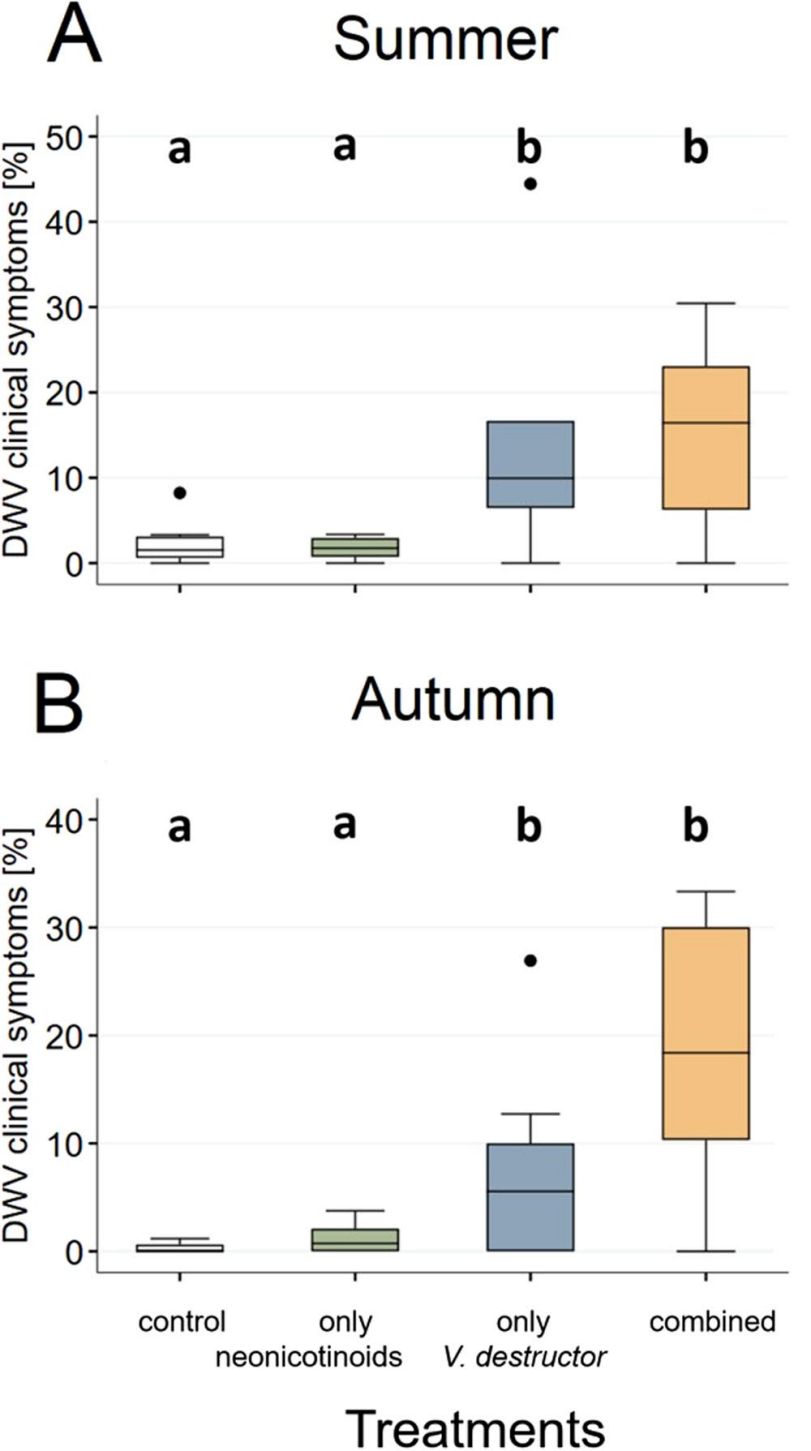
Proportion of honeybee (*Apis mellifera*) workers exposed to neonicotinoid insecticides and/or *Varroa destructor* and showing clinical symptoms of Deformed wing virus (DWV). Measurements of DWV clinical symptom levels for each of the four treatment groups were generated by uncapping worker brood cells and investigating wing anatomy for each individual. The level of DWV clinical symptoms was then calculated by dividing the total number of observed workers with symptoms by the total number of uncapped brood cells. The boxplots show the inter-quartile-range (box), the median (black line within box), and outliers (dots). A significant difference (generalized linear mixed model, *P* < 0.05) between groups is indicated by different letters (a, b).

No significant differences in mite infestation levels obtained by either mite washes, bottom board counts or brood dissection were observed between neonicotinoid-exposed and control colonies, neither for summer nor autumn (Extended Data Tables 1 and 2; Extended Data Fig. 1). Similarly, no significant differences were observed for levels of DWV clinical symptoms between only neonicotinoid-exposed and control workers in summer and autumn (Fig. 1; Extended Data Table 3). In contrast, a significant increase in the proportion of workers with DWV clinical symptoms was observed for the *V*. *destructor* parasitism and combined treatment groups compared to controls in both seasons (Fig. 1; Extended Data Table 3). No significant difference was observed between *V*. *destructor* parasitism and combined treatment groups (Fig. 1; Extended Data Table 3).

### Worker emergence mass

**Figure 2:**
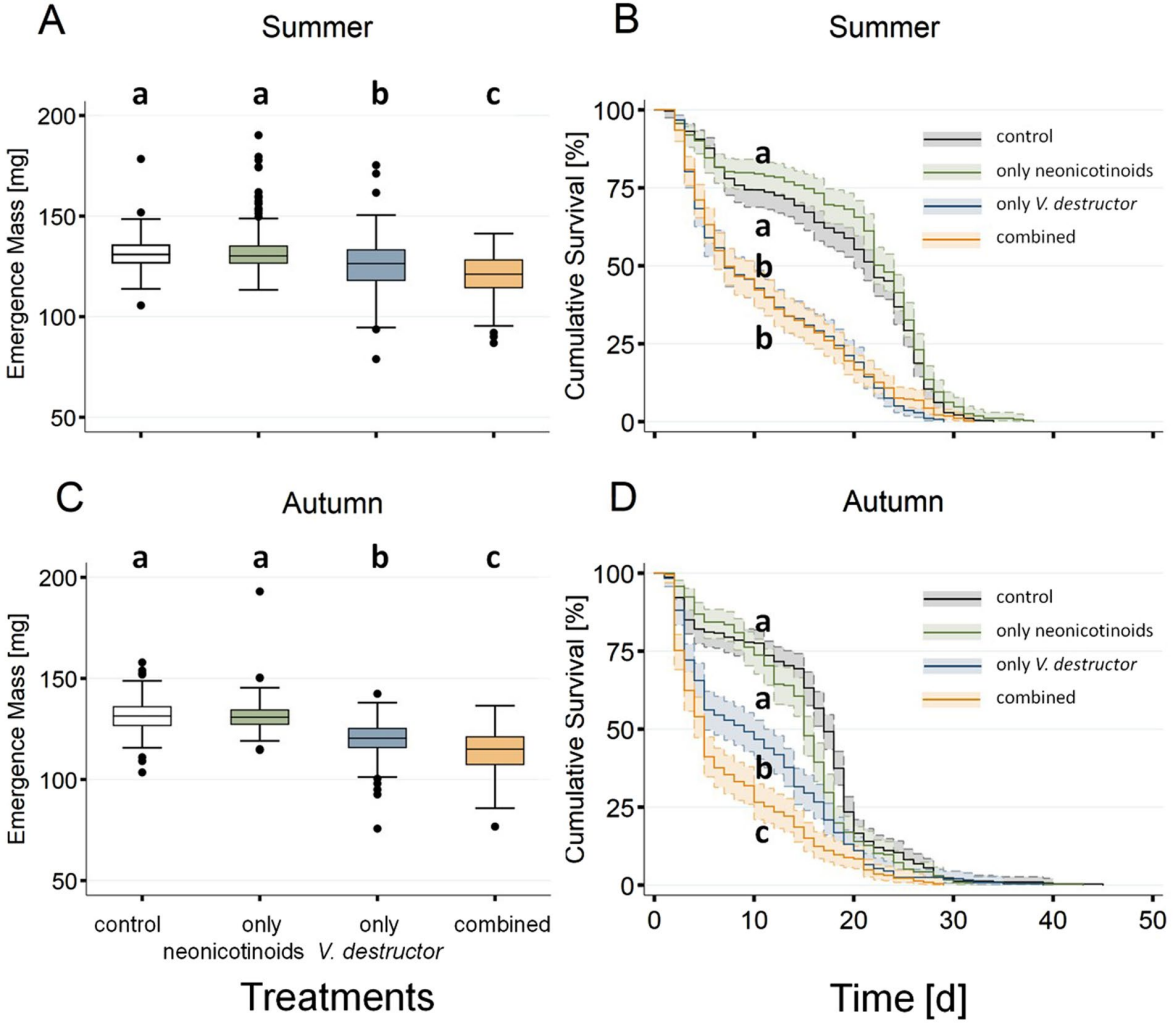
Effects of neonicotinoid insecticides and *Varroa destructor* on honeybee (*Apis mellifera*) worker emergence mass and survival. To obtain a fully crossed experimental design, honeybee colonies were exposed to neonicotinoids (4 ppb thiamethoxam and 2 ppb clothianidin) and to natural *V*. *destructor* infestation. Measurements of emergence mass were performed in summer and autumn. For each of the four treatment groups generated, body mass (1A, 1C) of 1430 workers originating from ten colonies was measured 24 h prior expected emergence date. The boxplots show the inter-quartile-range (box), the median (black line within box), and outliers (dots). Survival of the workers (N = 1105) kept in cages in groups of 10 was measured until all individuals died. The Kaplan-Meier curves show the survival over time of the four treatment groups (solid lines) as well as the 95% confidence intervals (CI) (shaded areas). A significant difference (generalized linear mixed model, *P* < 0.05) between groups is indicated by different letters (a, b, c).

There was no significant impact of only neonicotinoid treatments on worker emergence mass, in summer or in autumn (Fig. 2A,C; Extended Data Table 3). In contrast, a significant negative effect of *V*. *destructor* parasitism on worker emergence mass was observed in both summer and autumn (Fig. 2A,C; Extended Data Table 3). In both seasons, the decrease in emergence mass was strongest when parasitism was combined with neonicotinoids (Extended Data Table 4). Individuals exposed to only *V*. *destructor* parasitism or to both stressors in summer weighed less compared to controls, (4.4 and 8.1% reduction in median emergence mass, respectively; Extended Data Tables 4 and 5). A synergy between stressors was observed because the effect of combined stressor exposure (8.1%) was higher than the sum of individual effects (0 + 4.4% of only neonicotinoids and only *V*. *destructor* parasitism, respectively). Emergence mass in autumn decreased by 0.3% (only neonicotinoids), 8.6% (only *V*. *destructor* parasitism) and 13.2% (combined) on average (Extended Data Table 5). Therefore, synergistic effects were also present in autumn.

### Worker survival

Similar to emergence mass, exposure to only neonicotinoids did not yield a significant effect on worker survival in summer and autumn (Fig. 2B,D; Extended Data Table 3), whereas exposure to only *V*. *destructor* parasitism and combined stressors resulted in strong negative effects (Fig. 2B,D; Extended Data Table 4). In summer, survival of workers exposed to only *V*. *destructor* parasitism (measured as survival curve) was not significantly different compared to workers that were exposed to combined stressors, and resulted in a small increase in hazard rate (HR) of ~153 and ~122% compared to controls, respectively (Fig. 2B, Extended Data Fig. 2A, Extended Data Table 6). In autumn, a significant decrease in the survival of the workers exposed to the combined treatment with respect to only *V*. *destructor* parasitism was observed (Fig. 2B,D; Extended Data Table 3). The HR for only *V*. *destructor* parasitism and combined stressors in autumn was ~230 and ~272%, respectively (Fig. 2D, Extended Data Fig. 2B, Extended Data Table 6).

With respect to median survival, no significant difference was observed due to only neonicotinoids for both seasons (Extended Data Table 3). In summer, exposure to only *V*. *destructor* parasitism and to the combined stressors had a significantly larger effect by reducing the median survival of workers by 68% in both cases (Extended Data Table 5). Since the observed magnitude of the effect of combined stressors is equal to that of the sum of the individual stressors (0 + 68% of only neonicotinoids and only *V*. *destructor* parasitism), there was no evidence of synergy in summer (Extended Data Table 5). Evidence for synergism was obtained in autumn, when the large detrimental effects of exposure to only *V*. *destructor* parasitism on median survival (47% decrease) were surpassed by those of the combined exposure with neonicotinoids (70% decrease, Extended Data Table 5).

### Seasonal effects

**Figure 3:**
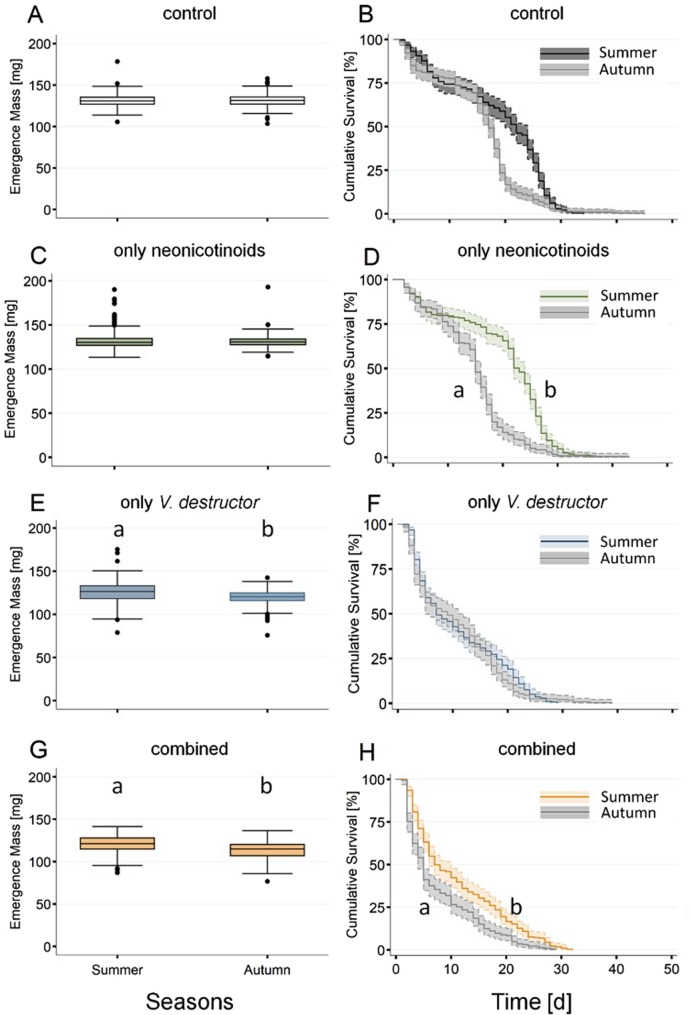
Seasonal effects of neonicotinoid insecticides and *Varroa destructor* on honeybee (*Apis mellifera*) worker emergence mass and survival. For each of the four treatment groups, body mass of 1425 workers originating from ten colonies was measured 24 h prior expected emergence date. The boxplots show the inter-quartile-range (box), the median (black line within box), and outliers (dots). Survival of the previously weighed workers (N = 1100) kept in cages in groups of 10 was measured until all individuals died. The Kaplan-Meier curves show the survival over time of the four treatment groups (solid lines) as well as the 95% CI (shaded areas). A significant difference (*P* < 0.05) between groups within the same cage assay is indicated by different letters (a, b).

Brood infestation rates revealed no significant seasonal effects for both control and neonicotinoid exposed colonies (Extended Data Table 3). Mite wash counts were significantly higher in autumn compared to summer for both control and neonicotinoid exposed colonies (Extended Data Table 3). Likewise, a significant seasonal effect was observed for bottom board counts for the control colonies, whereas no difference was seen for the neonicotinoid exposed colonies (Extended Data Table 3). No significant seasonal effect for the proportion of workers with DWV clinical symptoms was observed between summer and autumn for all treatment groups (Extended Data Table 3). Furthermore, no significant effects for emergence mass were observed between summer and autumn controls or for workers exposed to only neonicotinoids (Fig. 3A,C; Extended Data Table 3). In contrast, workers exposed to only *V*. *destructor* parasitism and to combined stressors were significantly lighter in autumn when compared to summer (Fig. 3E,G). For survival, no difference was observed between seasons for control workers (Fig. 3B) or workers exposed to only *V*. *destructor* parasitism (Fig. 3F), while seasonal effects were observed for the neonicotinoids and combined treatments (Fig. 3D,H) with survival being lower in autumn.

### Colony mortality and queen loss

There was no significant difference between control and neonicotinoid treated colonies in either winter mortality (one non-exposed, two neonicotinoid-exposed colonies: Chi-square test, χ^2^ = 0.56, df = 1, P = 0.45) or queen loss (one in non-exposed, two in neonicotinoid-exposed colonies, two treatment queens: Chi-square test, χ^2^ = 0.39, df = 1, P = 0.53).

## Discussion

Our data clearly show a significant negative synergistic effect of neonicotinoids and *V*. *destructor* mites on *A*. *mellifera* honeybee body mass and longevity. While negative synergism was observed for body mass in both summer and autumn, it was only observed for survival in autumn. This suggests a time-lag effect of previous neonicotinoid exposure that could contribute to elevated mortality of overwintering honeybee colonies. Even though colony mortality and queen loss were not significantly different between the treatment groups in our study, likely because we routinely treated our colonies against *V*. *destructor*, our data nevertheless suggest that the combined exposure to both parasitic mites and neonicotinoids increases the risk for honeybee colony mortality by negatively affecting the survival of winter honeybees.

Non-parasitized workers originating from colonies exposed to only neonicotinoids only, did not differ from the controls at any point, confirming earlier results^24^. In contrast, honeybee workers exposed to only *V*. *destructor* parasitism were consistently lighter and shorter-lived compared to the controls, which is also in line with previous studies^18,25^. The data also confirm the potent role of *V*. *destructor* as a virus vector^26^ due to the significantly higher proportions of bees with DWV clinical symptoms when infested by the mite. While laboratory data show positive interactions between viruses and insecticides^20^, the impact of insecticides on the occurrence of DWV clinical symptoms was not significant in our semi-field study (honeybees reared in field colonies but emerged in the laboratory). Had the virus-insecticide interface been a key factor, a significant effect on DWV clinical symptoms would have been observed. The underlying mechanism of the observed effects on mass and longevity is thus likely dominated by the mite-insecticide interface, with a varying impact of the mite-associated viruses. Indeed, this seems inevitable due to the highly variable nature of viruses^27^.

Interactions between stressors for honeybee health can in principle range from negative, over neutral and additive to synergistic ones^28^. Our study provides supporting evidence for the long-held hypothesis of synergism between neonicotinoids and *V*. *destructor*, constituting a possible mechanism for elevated losses of managed honeybee colonies^4^. This idea is supported by Blanken *et al*.^15^, who found a negative effect of this combination of stressors on a sublethal endpoint of individual bees (flight performance), while there was no effect of the stressors alone, hinting at an additive or synergistic interaction. Our study also showed that such synergism has lethal effects by affecting worker survival. A notable difference between the studies is the opposite effect of the combined stressors on the sublethal endpoint worker mass. Our study showed a negative effect while Blanken *et al*.^15^ showed a positive effect of the combined stressors on mass. This could be due to the different exposure routes of the neonicotinoids (pollen vs. sugar water, respectively) or to the different compound tested (thiamethoxam + clothianidin vs. imidacloprid, respectively). These same methodological differences might explain why a recent study by van Dooremalen *et al*.^14^ revealed that the combined exposure to a neonicotinoid and *V*. *destructor* did not yield significant negative effects on colony size or survival. In conclusion, it appears as if exposure route may be an important parameter to consider in risk assessment tests due to potential diverging consequences.

The synergism between neonicotinoids and *V*. *destructor* that we observed could be due to the mite infestation down-regulating the expression of cytochrome P450 genes in its host^13^. These genes play a crucial role in detoxification of xenobiotics, including neonicotinoids^13^. Even in the absence of a synergistic effect of these stressors on the expression of a gene from this family^13^, the effect of its down-regulation due to parasitism alone may lead to a negative synergetic effect on host survival. In absence of the insecticide, the likely reduced detoxification ability might be inconsequential, while in its presence, larger physiological effects than in the presence of the parasite alone might occur.

Synergism between neonicotinoids and *V*. *destructor* affected emergence mass in both summer and autumn, whereas it affected worker survival only in autumn. This decrease in survival 16 weeks post neonicotinoid colony exposure reveals a previously overlooked time lag effect of neonicotinoid exposure. The absence of significant neonicotinoid effects on survival of summer bees indicates that direct exposure to residues is unlikely to be responsible for the effect on workers in autumn. It was also not likely due to infestations by *V*. *destructor* alone since its effect on our endpoint measurements was constant over the seasons. Instead, this time lag effect most likely resulted from dysfunction within the colony environment (e.g., a reduction in nursing quality) due to the previous neonicotinoids exposure^29^. Time-lag effects could have also occurred because of an adaptive trait of honeybees to temperate regions. Under temperate climate, honeybee colonies produce two types of worker bees – short-lived summer bees in spring and early summer, and long-lived winter bees in late summer and autumn that are adapted to surviving this period of dearth^30^. The adaptation can include down-regulation of immune genes^31^, which might explain why susceptibility to both individual mite and combined stressors increased in autumn. The previously reported reduced longevity of winter bees exposed to *V*. *destructor* mites alone^32^ could be amplified by the here reported negative synergistic interactions between mites and neonicotinoids. Because honeybee colonies must produce sufficient quantities of long-living winter bees to survive^33^, the observed negative synergistic effects on individual winter honeybee longevity are most likely strongly compromising colony survivorship. Our results therefore imply that synergistic effects between these nearly ubiquitously occurring stressors are likely to contribute to honeybee colony losses over winter.

By providing essential pollination services to agriculture, managed honeybees are often exposed to agrochemicals. Given the ubiquitous nature of *V*. *destructor* mites potentially interacting with any agrochemical with a similar mode of action to neonicotinoids, it appears crucial to mitigate the synergistic interactions between such stressors. A reduction of honeybee colony exposure to agrochemicals, combined with more efficient and sustainable *V*. *destructor* solutions^34–37^, appears sensible in light of our results. In addition, investigating possible interactions between other common stressors of honeybees seem essential for mitigating *A*. *mellifera* colony mortality (e.g., between insecticides and malnutrition). This study highlights the possible important role of interactions between an invasive parasite and neonicotinoids in many regions of the world. However, current insecticide risk assessments largely ignore interactions between stressors and thus only provide a fragmentary evaluation of their possible effects^38^. Future efforts by risk assessors should therefore include possible interactions and time lag effects between agrochemicals and other stressors to safeguard honeybees.

## Material and Methods

### Set-up

The study was conducted in Bern, Switzerland between April 2014 and May 2015. We employed 20 local honeybee colonies, which were considered healthy based on *V*. *destructor* infestation levels and the absence of any obvious clinical symptoms of diseases based on visual inspections^39^. These colonies were established at the beginning of the experiment using shook swarms (one laying sister queen, ∼1.8 kg workers) and five Dadant frames equipped with organic worker cell wax foundation. Colonies were maintained by local Best Management Practices that included autumn and winter *V*. *destructor* mite management using formic (FAM dispenser, 70%) and oxalic (2.7%) acids^7^. Mite infestation levels were quantified for each colony (29 August 2014 & 31 October 2014) using the soapy water wash and sticky bottom board methods^39^ and individual worker brood cell infestations^39^.

### Neonicotinoid exposure

Colonies were provided with 100 g pollen paste [55% irradiated honeybee corbicular pollen, 5% brewer’s yeast, and 40% sucrose solution (60% w/v)] on top of the frames on a daily basis, and concurrently fitted with entrance pollen traps to promote feeding on the provided paste^22,40,41^. Colonies were randomly allocated to one of two treatments (neonicotinoid or no neonicotinoid) in mid May 2014. Pollen paste fed to the colonies belonging to the neonicotinoid treatment contained 4.0 ppb thiamethoxam and 2 ppb of its break-down product clothianidin (both Sigma-Aldrich), which represent field-realistic concentrations of the chemicals found in plant pollen^42^. For both neonicotinoid and control colonies roughly 90 kg of pollen paste were made. The patties were made in three individual batches by adding the appropriate amount of corbicular pollen, sugar powder and honey, as well as a neonicotinoid solution for the pesticide treatment group. After homogenization, a random sample of pollen paste was taken from each batch for residue analysis. The provided residue levels derive from taking the average value of the three random pollen paste samples from both treatment groups. Pollen patties were stored at −24 °C until they were fed to colonies. The applied concentrations were confirmed (3.9 ppb thiamethoxam and 1.9 ppb clothianidin in neonicotinoid insecticide patties; below limit of quantification for thiamethoxam (less than 0.02 ppb) and clothianidin (less than 0.08 ppb) in control patties) by the French National Centre for Scientific Research using ultra-high performance liquid chromatography-tandem mass spectrometry (UHPLC-MS/MS). Pollen paste feeding occurred for 42 days to expose colonies for at least two complete worker brood cycles. This ensured that the obtained experimental workers were also reared by workers exposed during their development.

### Experimental workers

For all laboratory cage assays, the queen of each colony was restricted for two days to a worker brood comb built by adult workers in the respective colony during treatment exposure. This resulted in known age-cohorts of workers brood^43^. The experimental frames remained in the respective colonies until ∼48 h prior to adult emergence, when they were transferred to a laboratory incubator maintained at 34.5 °C, 60% RH in complete darkness^43^. One-day later (i.e., one day before adult emergence) several hundred cells per colony were carefully and individually uncapped to obtain the required number of experimental workers. Each worker was visually examined to estimate appropriate cohort age^41^, mite infestations^39^, and clinical symptoms of disease^44^. The proportion of individuals showing clinical symptoms of DWV (crippled wings and/or shortened abdomens^21,45^) were recorded and used as a proxy of virus levels^45^ as well as a predictive marker of colony mortality^21^. Individuals displaying deformed wings were excluded. Each selected worker was weighed to the nearest 0.1 mg using an analytic scale (Mettler Toledo AT400), and then assigned to one of the four treatment groups based on their previous colony-level exposure to neonicotinoids and individual *V*. *destructor* parasitation status*:* 1. No neonicotinoids/No *V*. *destructor* parasitism (control), 2. No neonicotinoids/Yes *V*. *destructor* parasitism (only *V*. *destructor*), 3. Yes neonicotinoids/No *V*. *destructor* parasitism (only neonicotinoids), 4. Yes neonicotinoids/Yes *V*. *destructor* parasitism (combined)). Each treatment group consisted of 30 hoarding cages (80 cm³; 3 cages per treatment colony) that each contained 10 workers^43^. Cages were maintained in complete darkness at 30 °C and ~60% RH^43^, and provided 50% (w/v) sucrose solution *ad libitum*. Every 24 h, food was replaced, worker mortality was recorded, and dead honeybees were removed. Each cage assay was terminated when the last worker died.

Honeybee worker health was evaluated by measuring both sublethal (emergence mass) and lethal (survival) health indices, because *V*. *destructor* causes both^7^. This was required although the tested insecticide concentrations are believed to cause sublethal effects only, because the potential interactions with parasitism may be lethal.

### Timing

Two hoarding cage assays were performed, starting on 29 June 2014 (summer assay) and 21 September 2014 (autumn assay). These dates corresponded to 6.5 and 16 weeks post initial neonicotinoid exposure and were used to evaluate immediate effects after exposure (summer assay), as well as time-lag effects in autumn (autumn assay).

### Colony parameters

The colony strength parameters were assessed using the Liebefeld estimation method that visually quantified bees, capped and uncapped brood, honey, and pollen in each individual colony^46,47^. Colony strength variables (i.e., total bees, total capped brood surface [cm²], total uncapped brood surface [cm²], total honey surface [cm²], and total pollen surface [cm²]), were first evaluated as percent scores ranging from 0 to 100 of frame coverage. Percent coverage was then converted into absolute values of area [cm²] (e.g., total capped brood surface [cm²]) or weight [kg] (e.g., honey [kg])^47^. Assessments were carried out six weeks (23.06.2014) and 12 weeks (06.08.2014) post initial treatment exposure.

Bottom board counts, as well as soapy mite wash counts, were performed to determine colony level infestation rates of *V*. *destructor* mites^7,39^ between control and neonicotinoid insecticide exposed treatments for both seasons. In addition, *V*. *destructor* brood infestation rates (brood mites)^7^ were determined during uncapping of the experimental frames (see above) by dividing the total mite-parasitized uncapped cells by the overall total uncapped cells per colony.

Any possible seasonal effects were determined by comparing all three estimates of *V*. *destructor* infestation (brood, bottom board, soapy wash)^39^ and DWV clinical symptom levels between summer and autumn.

Colony mortality and queen loss were recorded throughout the entire experiment.

### Statistical analyses

Treatment and seasonal differences were assessed using generalized linear mixed (regression) models (GLMM) fitted using STATA15^48^. The treatment at the colony level was exposure to neonicotinoids or not. At the individual bee level, we had four treatment groups based on their previous colony-level exposure to neonicotinoids and individual *V*. *destructor* parasitism status 1. No neonicotinoids/No *V*. *destructor* parasitism (control), 2. No neonicotinoids/Yes *V*. *destructor* parasitism (only *V*. *destructor*), 3. Yes neonicotinoids/No *V*. *destructor* parasitism (only neonicotinoids), 4. Yes neonicotinoids/Yes *V*. *destructor* parasitism (combined). Treatment was included as the fixed explanatory variable, where applicable (see Extended Data Table 1). The colony and cage identification numbers were included as random effects to take into account the clustering effects due to every individual’s original colony and our experimental set up in cages^49^. Therefore, individual workers represented experimental units. In addition, a goodness of fit of the models was assessed by the analysis of residuals, with the STATA function predict [option deviance]. All figures were created using STATA15.

Count data variables (e.g., total bees, bottom board counts, and mite wash counts) were fitted to negative binomial models using the STATA15 function menbreg^50^. Total capped brood [cm²], total honey [kg], pollen patty consumption [g] or emergence mass [mg]) were modeled with GLMM of the Gaussian or Gamma family (depending on the analysis of residuals) using the STATA15 function meglm. Instead of transforming the outcome varaibles, we opted for the Gamma family that provided good fits (normality of the residuals).

Proportion of mite-infested brood and workers showing DWV clinical symptoms were recorded as a score ranging from 0 to 100% and subsequently an ordered logistic model was applied using the function meologit^51^. Multiple pairwise comparisons (Bonferroni test) for mite infestation levels and emergence mass were obtained by using the mcompare(bonferroni) function^52^. Chi-square was used to assess for significant differences between control and neonicotinoid insecticide exposed colony mortality and queen losses.

Worker survival data for all cage assays and treatment groups were fit using the mestreg function for multilevel survival models^49,53^. Median survival was calculated as the 50^th^ percentile of the survival time^25^. Furthermore, two different models for adjusting survivor functions for the effects of covariates were used: the proportional hazards (PH) model and the accelerated failure time (AFT) model^54,55^ (Extended Data Table 6). Both models enable the calculation of a regression coefficient, in our case the hazard rate of a specific treatment group compared to another treatment group (i.e., control). Survival curves (Kaplan Meier plots) and smooth estimated hazard rate plots with 95% CI were visually inspected to determine the appropriate model and are presented in the Extended Data Fig. 2. If the survival curves declined parallel to one another at the same ratio, the PH model (with a Weibull distribution) was applied; if curves diverged, with one showing an increased decline, an AFT model was applied. As its name suggests, the AFT model accounts for an accelerated decrease in the survival rate of the considered treatment with respect to control. Due to the different parameterizations, the coefficients of both models are not directly comparable and conversion is needed. In STATA, the PH model directly calculates a hazard ratio; however, AFT calculates a coefficient’s factor which can then be exponentiated and converted into a hazard ratio^56^. The relationship between the coefficients of both models (using the PH model with a Weibull distribution) was:

$$Hazard\,Rati{o}_{PH}={\exp }(\,-\,\exp ({ln}\,p)\ast {\beta }_{AFT})$$

where *β*_*AFT*_ is the regression coefficient of the AFT model and p denotes the ancillary parameter (which is estimated in logarithmic metric and displayed in STATA output as \ln_p). The hazard ratios can be interpreted as ‘conditional hazard ratios’ that is conditional on the random effects. A hazard ratio of 1.01 means 1.01 × 100% = 101%, that is a 1% increase in the hazard for each unit of the explanatory variable. In addition, multiple pairwise comparisons (Bonferroni test) for worker survival amongst the different groups were obtained by using the mcompare(bonferroni) function^56,57^. Whenever possible, every three-level model was compared with its single-level model counterpart using a Likelihood ratio (LR) test^58^. Likelihood ratio (LR) tests, which did not rely on the assumption of asymptotic normal sampling distributions, were used to demonstrate which model best fit the data.

### Interactions

To further identify potential synergistic interactions between *V*. *destructor* and neonicotinoids, we employed an additive effects framework^28^. In this model, synergism and antagonism occur when the combined effect of multiple stressors is greater (synergism) than the sum or smaller (antagonism) than the sum of effects elicited by individual stressors^59^. Interactive stress effects on emergence body mass and survival were calculated as the percent difference in treated groups relative to controls, whereby the mean emergence mass [g] and median survival [d] were used for the calculations.

## Supplementary information

Supplementary Information

## Data Availability

The complete raw data can be found on the Dryad repository (10.5061/dryad.r886083).
