# Supplementary material for: Neonicotinoids and ectoparasitic mites synergistically impact honeybees
Source: Sci Rep. 2019 Jun 4;9:8159. doi: 10.1038/s41598-019-44207-1 (PMC6547850; doi:10.1038/s41598-019-44207-1)
Supplement: Supplementary file 1 — Supplementary Information [file 41598_2019_44207_MOESM1_ESM.docx]

**Supplementary Information**

**Neonicotinoids and ectoparasitic mites synergistically impact honeybees**

Lars Straub^1,2*^, Geoffrey R. Williams^1,2,3*^, Beatriz Vidondo^4*^, Kitiphong Khongphinitbunjong^5,6^, Gina Retschnig^1^, Annette Schneeberger^1^, Panuwan Chantawannakul^5,7^, Vincent Dietemann^3,8^, Peter Neumann^1,2,5^

^1^ Institute of Bee Health, Vetsuisse Faculty, University of Bern, Bern, Switzerland.

^2^ Swiss Bee Research Centre, Agroscope, Bern, Switzerland.

^3^ Department of Entomology and Plant Pathology, Auburn University, Auburn, AL, USA.

^4^ Veterinary Public Health Institute, Vetsuisse Faculty, University of Bern, Bern, Switzerland

^5^ Bee Protection Laboratory, Department of Biology, Faculty of Science, Chiang Mai University, Chiang Mai, Thailand.

^6^ School of Science, Mae Fah Luang University, Chiang Rai, Thailand.

^7^Environmental Science Research Center, Faculty of Science, Chiang Mai University, Chiang Mai, 50200, Thailand

^8^Department of Ecology and Evolution, University of Lausanne, 1015 Lausanne, Switzerland

*Contributed equally

**
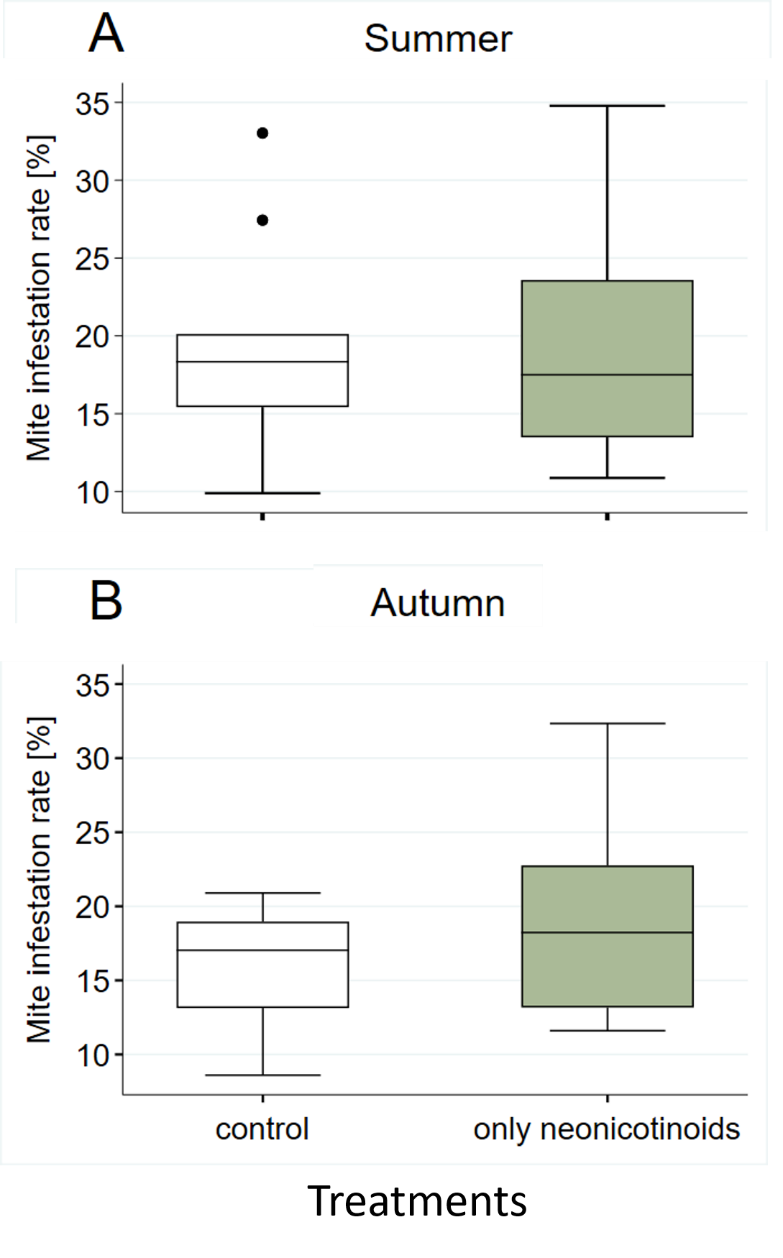
**

**Extended Data Figure 1 | Proportion of honeybee (*Apis mellifera*) workers brood cells infested with the ectoparasitic mite, *Varroa destructor.*** Measurements of mite infestation rates for both control and neonicotinoid exposed treatments were generated by uncapping worker brood cells and determining the presence of *V. destructor* for each individual cell. The infestation rate was then calculated by dividing the total number of infested worker cells by the total number of uncapped brood cells. No significant differences were observed for either summer (A) or autumn (B). The boxplots show the inter-quartile-range (box), the median (black line within box), and outliers (dots).

**
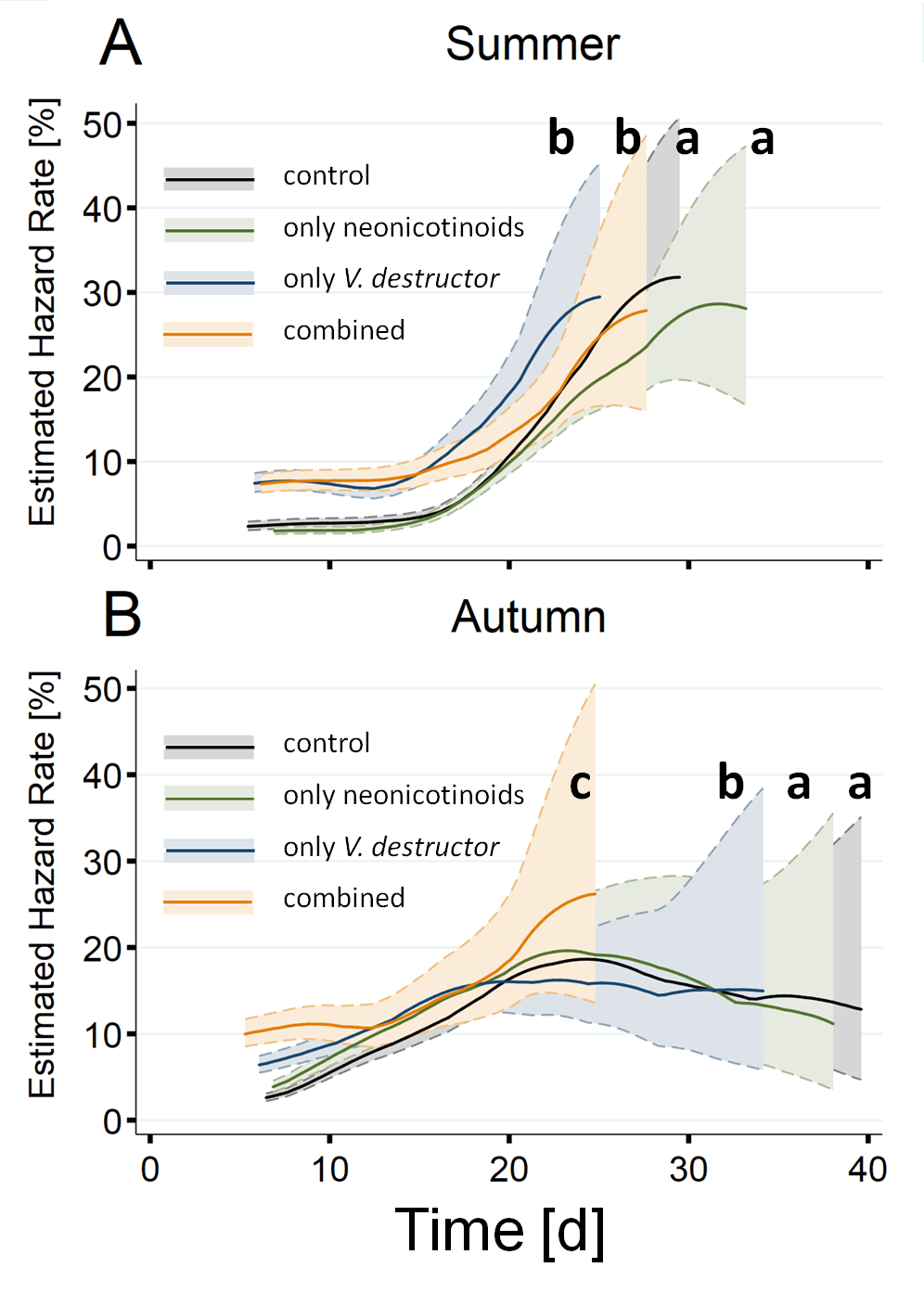
**

**Extended Data Figure 2 | Effects of neonicotinoids and *Varroa destructor* on honeybee (*Apis mellifera*) worker survival hazard rates.** To obtain a fully crossed experimental design, honeybee colonies were exposed to neonicotinoids (4 ppb thiamethoxam and 2 ppb clothianidin) and to natural *V. destructor* infestation alone or in combination. Measurements of survival were performed in summer and autumn. For each of the four treatment groups, survival of 1100 workers kept in cages in groups of 10 and originating from ten colonies was measured until all individuals died. Based on the censored survival models (GLMM) the estimated hazard rates were calculated. The estimated hazard rate curves show the four treatment groups (solid line) as well as the 95 % CI (shaded area) over time. A significant difference (generalized linear mixed model, *P* < 0.05) between treatment groups is indicated by different letters (a, b, c).

**Extended Data Table 1 | Summary of statistical methods and results for the effects of neonicotinoids and *Varroa destructor* on honeybee (*Apis mellifera*) worker emergence mass, survival and DWV infection levels.** The STATA15 functions applied for each season were fit for all measured variables (e.g., emergence mass [mg], survival [d], total honey [kg]). Furthermore, the respective level of the applied generalized linear model, STATA15 function, as well as the calculated P-values are presented. One-level models only contain fixed effects (such as treatment groups). Two-level models contain one random effect (colony), and three-level models contain two random effects (colony and cage).

**Extended Data Table 2 | Summary of counted *Varroa destructor* mites from bottom board and soapy mite washes from both summer (29.08.2014) and autumn (31.10.2014).**

**Extended Data Table 3 | P-values from the generalized linear mixed models (GLMMs).** In case of multiple comparisons, a post-hoc Bonferroni test was applied to determine significant differences among all pairs of treatment groups for all variables. P-values remaining significant after correction are indicated with an *.

**Extended Data Table 4 | Summary of results of the effects of neonicotinoid insecticides and *Varroa destructor* on honeybee (*Apis mellifera*) worker for all outcome variables.**

**Extended Data Table 5 | Summary of interactions between neonicotinoid insecticides and *Varroa destructor* on honeybee (*Apis mellifera*) worker emergence mass and survival.** Based upon an additive effects model, stressor effects were calculated as the percent difference in treatments relative to controls, whereby the mean emergence mass [g] and median longevity [d] were used for the calculations. Synergistic and antagonistic effects were determined by comparing the sum of individual effects with the effects of the combined stressors. Synergism occurs when the combined effect of multiple stressors is greater than the sum of effects elicited by individual stressors.

**Extended Data Table 6 | Summary of statistical results obtained from multilevel survival models for the effects of neonicotinoids and *Varroa destructor* on honeybee (*Apis mellifera*) worker survival.** A three-level survival model was applied to determine whether the individual or combined stressors significantly affected worker survival when compared to the reference group (control) for both summer and autumn. Two models for adjusting survivor functions for the effects of covariates were used depending on the progression of the curves: the proportional hazards (PH) model and the accelerated failure time (AFT) model. Both models enabled the calculation of a regression coefficient which subsequently enabled the calculation of the hazard ratio [%] of each specific treatment group in comparison to the control. Furthermore, the 95% confidence intervals (CI), as well as the logarithmic metric (Ln_p), are presented for each treatment group

**Extended Table 7 | Summary of statistical results obtained from the generalized linear mixed model (GLMM) for the effects of neonicotinoids and *Varroa destructor* on honeybee (*Apis mellifera*) worker emergence mass.** A two-level GLMM was applied to determine whether the individual or combined stressors significantly affected worker mass in summer and autumn. All obtained *P*-values and the corresponding coefficients and robust standard errors (Std. Err.), as well as the lower and upper 95% confidence intervals (CI), are presented.
